# Association between rs6759298 and Ankylosing Spondylitis in Iranian Population

**Published:** 2018

**Authors:** Mahdi Mahmoudi, Masoud Garshasbi, Amir Ashraf-Ganjouei, Ali Javinani, Mahdi Vojdanian, Masoumeh Saafi, Nooshin Ahmadzadeh, Ahmadreza Jamshidi

**Affiliations:** 1. Rheumatology Research Center, Tehran University of Medical Sciences, Tehran, Iran; 2. Department of Medical Genetics, Faculty of Medical Sciences, Tarbiat Modares University, Tehran, Iran; 3. Students’ Scientific Research Center, Tehran University of Medical Sciences, Tehran, Iran; 4. Department of Genetics, Islamic Azad University, Tabriz Branch, Tabriz, Iran

**Keywords:** Ankylosing spondylitis, HLA-B27, Iran

## Abstract

**Background:**

Ankylosing Spondylitis (AS) is a chronic autoinflammatory Spondyloarthropathy (SpA) which is characterized by sacroiliitis, which progresses to the axial skeleton. It seems that non-Human Leukocyte Antigen (HLA) and also HLA-B27 are associated with the susceptibility and pathogenesis of the disease. The recent Ge-nome-Wide Association Studies (GWASs) have reported intergenic rs6759298 to be associated with AS etiology. The aim of this study was investigation of the rs6759298 polymorphism in Iranian AS patients. In addition, probable correlations with clinical indices and manifestations were considered.

**Methods:**

This study included 403 patients with AS. The control group consisted of 506 healthy individuals who were matched for sex, age, and ethnicity with AS group. Genotyping of rs6759298 was determined using the Amplification-Refractory Mutation System-Polymerase Chain Reaction (ARMS-PCR).

**Results:**

The GG genotype and G allele were found to be significantly more prevalent in the patient group in comparison to the control group [(p=2×10^−6^ and 7.44×10^−9^; OR (95% CI) =2.16 (1.56–2.98) and 1.73 (1.43–2.08)], respectively.

**Conclusion:**

No associations were found between patients with three genotypes and any disease manifestations or clinical indices. This investigation confirmed a highly significant association of rs6759298 with disease susceptibility, with no effect on disease progress or clinical presentations. Since rs6759298 belongs to the *2p15* gene desert, further studies would elucidate the exact role of this polymorphism in the pathogenesis of AS.

## Introduction

Ankylosing Spondylitis (AS) is the most prevalent disease among the Spondyloarthritis (SpAs) family. SpAs are a group of disorders characterized by positive Human Leukocyte Antigene-B27 (HLA-B27) and in-flammation of the axial skeleton or peripheral joints. SpAs are categorized based on their associated disorders and the anatomical locations involved. Co-occurrence with psoriasis and Inflammatory Bowel Disease (IBD) proposes that these disorders and SpAs may share a common pathophysiologic pathway. On the other hand, reactive arthritis as another member of the SpA group suggests that microbial factors may also play a role 1.

AS is an autoinflammatory SpA, which is characterized by progressive sacroiliitis to the axial skeleton. Osteophyte formation and spine ankylosis are morbid manifestations, which reduce spine mobility and chest expansion. As opposed to most autoimmune diseases, AS is a male dominant disorder with a sex ratio of 3.8:1 2. Disease onset is characterized by inflammatory pain of the sacroiliac joint that can be detected using various imaging methods. HLA-B27 is present in 73% of Iranian patients and can therefore be used as a reliable auxiliary tool for AS diagnosis 3–5. Symptoms such as peripheral arthritis, enthesitis, and uveitis are the most common non-axial involvements seen in AS patients 6.

**Figure 1.**
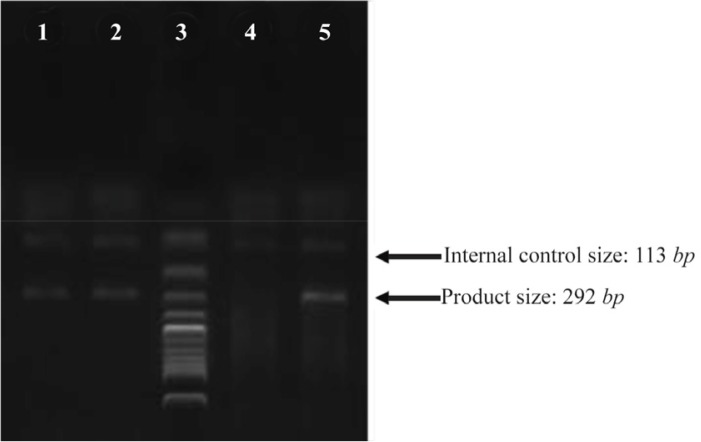
PCR products from various samples. Lane 3: ladder, lane 4: internal control only, lane 1, 2, 5: PCR products from three samples with C alleles.

Due to the prevalence and morbidity of AS, many studies have been performed to elucidate its precise pathogenesis. Similar to other non-infectious diseases, gene polymorphisms have been found to play a noticeable role, which has been confirmed by Single Nucleotide Polymorphism (SNP) analyses on genes such as Cytotoxic T-lymphocyte-associated Protein-4 (CTLA4), Programmed cell Death-1 (PD1) and Endoplasmic Reticulum Aminopeptidase1 (ERAP1) 7–9. Among these SNPs, HLA-B27 has the most reliable role, based on its significantly different prevalence between patients and healthy individuals. As an antigen presenting molecule, HLA-B27 has been attributed with several hypotheses, indicating the antigen processing/presenting defect in AS and its role in adaptive immunity. Recent hypotheses suggest a pathologic role for HLA-B27 in innate immunity and Endoplasmic Reticulum (ER) stress 10–12. These claims were challenged by explanations given for the function of ERAP1 in AS pathogenesis. ERAP1, a non-HLA protein, is involved in the antigen presenting process in the endoplasmic reticulum and its gene interaction with HLA-B27 has recently been proved 9,13. According to other gene polymorphism studies carried out in the Iranian population, Interleukin-1 Receptor (IL-1R) and PD1 were also suggested as non-HLA genes which may be associated with AS pathogenesis 7,9.Figure 1. PCR products from various samples. Lane 3: ladder, lane 4: internal control only, lane 1, 2, 5: PCR products from three samples with C alleles.

In addition to many SNPs in genes such as *CTLA4*, *PD1* and *ERAP1*, recent studies have shown that there is a strong association between few intragenic SNPs and AS in different populations. For instance, three SNPs in 2p15 locus (rs10865331, rs10865332, and rs4672503) and three SNPs at chromosome 21q22 (rs-2242944, rs2836878, and rs378108) achieved genome-wide significance with AS risk 14.

Genome-Wide Association Studies (GWASs) have shown a number of potentially functional intronic variants such as those in 2p15, 21q22 locus, and rs6759298 15,16. In this study, polymorphism of rs6759298 was analyzed in an Iranian AS cohort. Then, its probable associations with clinical manifestations and disease activity indices were analyzed.

## Materials and Methods

### Study population

This study was conducted on 403 AS patients from the outpatient rheumatology clinic of Rheumatology Research Center, Tehran University of Medical Sciences who fulfilled the 1984 modified New York criteria for classification of AS 17. They consisted of 89 female and 314 male individuals (3.52:1 ratio) with a mean age of 38.14 years (minimum 18 and maximum 75 years old, standard deviation (SD)=10.26). The control group for this study was composed of 506 age, sex, and ethnicity matched healthy individuals with a mean age of 36.6 years old and a 3.21:1 ratio of male to female individuals (386 males and 120 females, SD= 10.73). Clinical manifestations such as uveitis, aphthae, arthritis, enthesitis and clinical indices such as Bath Ankylosing Spondylitis Functional Index (BASFI), Bath Ankylosing Spondylitis Metrologic Index (BAS-MI), Bath Ankylosing Spondylitis Disease Activity Index (BASDAI), and Ankylosing Spondylitis Quality of Life (ASQOL) were evaluated. Peripheral blood collection began on November 2013 and finished on June 2015. Written informed consent forms were taken from all individuals and study protocol was approved by the Ethics Committee of Tehran University of Medical Sciences.

### DNA preparation and polymorphism analysis

Blood samples were collected in EDTA tubes and their leukocytes were used as a genomic DNA reservoir, with extraction using the standard phenol-chloroform method and subsequent storing at −20°*C* 18. rs-6759298 SNP was genotyped by the amplification refractory mutation system-polymerase chain reaction (ARMS-PCR) with a reverse primer sequence of (5′-TGGTGGTTCTGTAGGTAAATGG-3′) for the C al-lele, (5′-TGGTGGTTCTGTAGGTAAATGC-3′) for the G allele and (5′-GCCTTGTCAGATTCTTCAG-3′) for the forward primer sequence. Figure 1 shows PCR products from three samples with C allele.

PCR cycling for rs6759298 was carried out in three steps; A: initial denaturation at 95°*C* for 5 *min*, B: 10 cycles each of 30 *s* at 95°*C*, 30 *s* at 65.7°*C* and 60 *s* at 72°*C*, 25 cycles each of 30 *s* at 95°*C*, 30 *s* at 60.6°*C* and 60 *s* at 72°*C*, and C: final incubation at 95°*C* for 10 *min*. The PCR products were electrophoresed on 2% agarose gels and photographs were taken by the Vilber Loumat gel documentation instrument.

**Figure 2.**
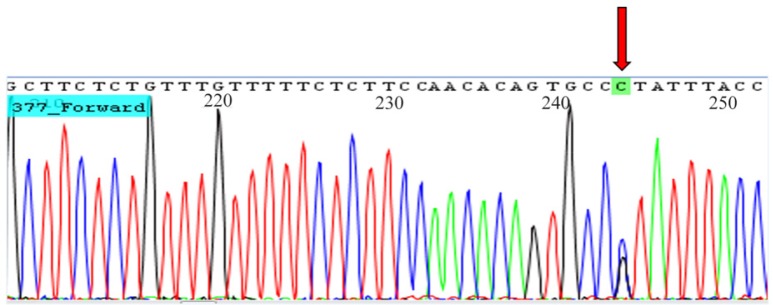
Chromatogram shows heterozygote GC genotype for rs6759298.

Direct Sanger sequencing of three samples with CC, CG, and GG genotypes were performed by 3730xl DNA Analyzer (Applied Biosystems) using big dye terminator (Figure 2).

### Statistical analysis

The control group was tested to meet the Hardy-Weinberg Equilibrium (HWE). The allele/genotype and clinical manifestations associations were analyzed by chi-squares test with Odds Ratio (OR) and 95% Confidence Intervals (CI). Probable correlation of rs6759298 genotypes with indices was analyzed with one-way ANOVA. P-values were adjusted with the Benjamini-Hochberg method to control for False Discovery Rates (FDR). The SPSS for Windows software (version 19.0, IBM SPSS Inc., USA) was used to perform all analyses.

## Results

No significant deviation of rs6759298 genotype distribution from Hardy-Weinberg Equilibrium was observed in healthy controls. The associations between allele and genotype frequencies with disease susceptibility are shown in table 1. According to these results, GG genotype and G allele significantly increased the susceptibility to AS [p=2×10^−6^ and 7.44×10^−9^, OR (95% CI)=2.16 (1.56–2.98) and 1.73 (1.43–2.08), respectively].

**Table 1.** Alleles and genotypes distribution of rs6759298 polymorphism in Iranian AS patient and healthy individuals

**dbSNP**	**Allele genotypes**	**Control (N=506)**	**AS^1^ (N=403)**	**p-value**	**OR (95% CI)^2^**
**rs6759298**					
	C	611	378	-	-
	G	399	428	7.44×10^−9*^	1.73 (1.43–2.08)
	CC	186	90	-	-
	GC	241	198	0.65	1.06 (0.82–1.38)
	GG	79	115	2×10^−6*^	2.16 (1.56–2.98)
**HWE^3^**		0.95			

1) Ankylosing Spondylitis;

2) Odd Ratio with 95% of Confidence Interval;

3) Hardy-Weinberg equilibrium;

* Significant p-values.

In addition to the preceding results, association of the rs6759298 genotypes and clinical manifestations is shown in table 2. Among a wide range of signs and symptoms, the most prevalent items were analyzed: enthesitis, arthritis, uveitis, and oral aphthae. Correlation of the mentioned genotypes with clinical indices is also included in table 2. No associations were discovered in this survey.

**Table 2.** Association of rs6759298 polymorphism with clinical manifestations and indices

**Clinical manifestation**	**GG**	**GC**	**CC**	**p-value**	**p-value^1^**	**Clinical index**	**GG mean**	**GC mean**	**CC mean**	**p-value**	**p-value^1^**
**Uveitis**	13	19	17	0.263	0.477	BASFI^2^	4.239	3.608	3.919	0.216	0.288
**Aphthae**	23	53	36	0.358	0.477	BASMI^3^	4.236	3.974	3.926	0.520	0.520
**Arthritis**	34	73	46	0.758	0.758	BASDAI^4^	5.292	4.573	4.413	0.066	0.264
**Enthesitis**	46	114	65	0.221	0.477	ASQOL^5^	8.702	7.206	7.764	0.136	0.272

1) FDR-adjusted P-value for multiple testing using the Benjamini–Hochberg method;

2) Bath Ankylosing Spondylitis Functional Index;

3) Bath Ankylosing Spondylitis Metrologic Index;

4) Bath Ankylosing Spondylitis Disease Activity Index;

5) Ankylosing Spondylitis Quality of Life.

## Discussion

By conducting several GWASs in AS patients over the past few years, researchers have identified a number of potentially functional intronic variants 14,19. However, it is not yet known whether such SNPs have direct functional significance or are simply in Linkage Disequilibrium (LD) with another functional SNP 15. In the current study, one of the most significant intergenic SNPs associated with AS was examined for the first time among the Iranian population.

rs6759298 SNP belongs to the *2p15* gene desert, containing some other SNPs associated with AS 16. According to HapMap genotype data (HapMap #27, on NCBI B36 assembly), rs6759298 is not included within the LD blocks of its neighboring genes. Therefore, rs-6759298 association with AS is unlikely due to an adjacent functional gene only.

The closest protein-coding genes to rs6759298 are *B3GNT2* genes (UDP-GlcNAc: betaGal beta-1,3-N acetylglucosaminyltransferase) which codify for a type II transmembrane protein involved in the biosynthesis of poly-N-acetyllactosamine chains, COMMD1 (copper metabolism domain containing, coding a regulator of copper homeostasis, sodium uptake, and NF-kappa-B signaling, and finally TMEM17 (transmembrane protein 17), coding a transmembrane component of a complex, which is required for ciliogenesis and sonic hedgehog/SHH signaling 20–22. Although *B3GNT2* that encodes UDP-GlcNAc is the closest gene to rs6759298, there is not any known immunological function for UDP-GlcNAc. Then, our variant was tested to predict probable functional consequences (ENSR 00001045107). Results suggested that rs6759298 is located within a promoter flanking region which has unknown regulatory functions and is active in the GM12878, HSMM tube, and K562 cell lines 14,23. Therefore, it is assumed that it could be a proximal promoter element for an AS-associated gene in the same region which has not yet been discovered. Another explanation is that it could be part of a long-range regulatory element involved in the transcription regulation of the aforementioned genes.

## Conclusion

To the best of our knowledge, this is the first study that provides evidence of association of the rs6759298 SNP with AS patients in the Iranian population. However, further molecular studies are necessary to demonstrate the exact role of such variants in the pathogenesis of AS.
